# Factors Associated with Lung Cancer Patients Refusing Treatment and Their Survival: A National Cohort Study under a Universal Health Insurance in Taiwan

**DOI:** 10.1371/journal.pone.0101731

**Published:** 2014-07-07

**Authors:** Hsiu-Ling Huang, Pei-Tseng Kung, Chang-Fang Chiu, Yueh-Hsin Wang, Wen-Chen Tsai

**Affiliations:** 1 Department of Health Services Administration, China Medical University, Taichung, Taiwan, R.O.C; 2 Hospital and Social Welfare Organizations Administration Commission, Ministry of Health and Welfare, Taiwan, R.O.C; 3 Department of Healthcare Administration, Asia University, Taichung, Taiwan, R.O.C; 4 Department of Internal Medicine, China Medical University, Taichung, Taiwan, R.O.C; H. Lee Moffitt Cancer Center, United States of America

## Abstract

**Background:**

Lung cancer is the leading cause mortality among all cancers in Taiwan. Although Taiwan offers National Health Insurance (NHI), occasionally, patients refuse treatment. This study examined the patient characteristics and factors associated with lung cancer patients refusing cancer treatment in four months after cancer diagnosed and compared the survival differences between treated and non-treated patients.

**Methods:**

The study included 38584 newly diagnosed lung cancer patients between 2004 and 2008, collected from the Taiwan Cancer Registry, which was linked with NHI research database and Cause of Death data set. Logistic regression was conducted to analyze factors associated with treatment refusal. The Cox proportional hazards model was used to examine the effects of treatment and non-treatment on patient survival and the factors affecting non-treatment patient survival.

**Results:**

Among the newly diagnosed cancer patients, older adults, or those who had been diagnosed with other catastrophic illnesses, an increased pre-cancer Charlson Comorbidity Index (CCI) score, and advanced stage cancer exhibited an increased likelihood of refusing treatment. Compared with treated patients, non-treated patients showed an increased mortality risk of 2.09 folds. The 1-year survival rate of treated patients (53.32%) was greater than that of non-treated patients (21.44%). Among the non-treated patients, those who were older, resided in lowly urbanized areas, had other catastrophic illnesses, a CCI score of ≥4, advanced cancer, or had received a diagnosis from a private hospital exhibited an increased mortality risk.

**Conclusions:**

Despite Taiwan's NHI system, some lung cancer patients choose not to receive cancer treatment and the mortality rate for non-treated patients is significantly higher than that of patients who undergo treatment. Therefore, to increase the survival rate of cancer patients, treatment refusal should be addressed.

## Introduction

Lung cancer is the leading cause of death among all cancer types, accounting for 13% of cancer mortality worldwide [1]. In American, lung cancer is expected to account for between 26% and 28% of all cancer deaths in 2013 [2]. According to World Health Organization (WHO), early detection of cancer and provision of appropriate treatment intervention increase cancer recovery rates. However, early stage lung cancer is difficult to diagnose. For non-small cell lung cancer (NSCLC) the proportion with metastatic disease (TNM stage IV) was 46.8% in Sweden and 48.4% in UK, and the NSCLC patients' 1-year survival was 46% in Sweden and 30% in UK [3]. Worldwide, estimated 5-year survival in NSCLC is only 16%[2].

In Taiwan, studies have indicated that lung cancer was responsible for 19.7% of cancer mortality in 2012 [4], and only approximately 20% of early stage lung cancer is diagnosed [5]. Overall the 1-year survival rate was 46.1% [6], and the 5-year survival rate were 15.9%, with a median survival of 13.2 months [7]. Medical advances have progressively increased lung cancer survival rates and the implementation of the National Health Insurance (NHI) program in Taiwan has substantially reduced barriers to access health care. However, each year, some patients refuse to accept treatment. Therefore, the factors underlying treatment refusal are worth examining.

The methods of lung cancer treatment consisted of chemotherapy, surgery, radiation, targeted therapy, or combined therapies [8], [9]. Cancer treatment requires a prolonged process during which patients continuously experience uncertainties regarding curability and mortality [10]. When patients lose control of their bodies and dignity, they are prone to becoming depressed and may subsequently choose to refuse or avoid medical treatment [11], [12]. Studies have indicated that among cancer patients, elderly adults are likely to refuse treatment or only receive partial treatment [13], [14]. Studies on treating patients with non-small cell lung cancer have found that not undergoing surgery is associated with a low socioeconomic level and single status [15].

Taiwan's NHI is a universal health insurance program that was implemented in March 1995. The NHI program provided a comprehensive benefit package that covers preventive and medical services, dental services, prescription medications, home nurse visits, and Chinese medicine treatment [16]. By the end of 2012, 99.85 percent of the total eligible population had enrolled in the NHI [4]. Low-income patients are exempted from paying health insurance premiums and co-payments. Under the NHI system, cancer patients are also exempted from medical service co-payments for cancer treatment. Despite the NHI, a proportion of lung cancer patients remain unwilling to receive treatment [5]. Therefore, the purpose of this study was to explore the characteristics and survival rates of lung cancer patients who refused treatment and to examine the associated factors.

## Methods

### Study Population

We collected the data of newly diagnosed lung cancer patients (ICD-O codes C 339–349) from the Taiwan Cancer Registry between 2004 and 2008. The data of these cancer patients were linked with the National Health Insurance Research Database (NHIRD) and Cause of Death data that are provided by the Ministry of Health and Welfare to conduct analysis. Personal information was removed from the research data and this study was reviewed and approved by an institutional review board (IRB No.: CMUH102-REC3-076). The patients were tracked until the end of 2010.

### Study Variables

In this study, lung cancer was defined as having a primary lesion in the lungs. However, because of data registration limitations, small cell lung cancer patients were excluded. Refusal of lung cancer treatment was defined as not receiving typical Western medical treatment, such as surgery, radiation therapy, chemotherapy, chemo-radiation therapy, or targeted therapy for a minimum of 4 months after the day of diagnosis. In addition, patients who received only palliative care were excluded from the sample.

The present study defined the non-receiving treatment for a minimum of 4 months after the day of diagnosis as denying treatment, because the Taiwan Health Promotion Administration sets this definition based on cancer-related experts' suggestions for the Taiwan Cancer Registry. In addition, according to the study conducted by Tsai [6], the study used the Taiwan Cancer Registry Database and found if cancer patients did not receive the cancer-related treatments within four months after diagnosis, the patients barely received treatments. Tsai's study showed increases in patients receiving cancer-related treatment after four-month delayed treatment were less than 3.5 percent in lung cancer patients. Therefore, this study followed the definition in the Taiwan Cancer Registry managed by Taiwan Health Promotion Administration and defined denying treatment as the cancer patients not receiving any treatment within four months after cancer diagnosis.

Demographic data variables were sex, age at cancer diagnosis, urbanization level of residence area (overall 7 levels; Level 1 was the most urbanized), socioeconomic status (determined by the insured monthly salary and insurance category), and the accreditation level and ownership of the diagnosing hospital. Personal health status involved the cancer stage, pre-cancer comorbidity index [i.e. the Charlson Comorbidity Index (CCI) [17]], and the presence of other catastrophic illnesses.

### Statistical Analysis

Descriptive statistics were used to analyze the data of the newly diagnosed cancer patients between 2004 and 2008 who did not receive treatment. The analyzed data contained personal demographics and health status. The *t* test and chi-square test were used to examine the differences in personal demographics, health status, lung cancer severity, and diagnosing hospitals between treated and non-treated patients. Furthermore, to prevent a patient cluster effect whereby some patients were diagnosed at the same hospitals, the generalized estimating equation (GEE) was calculated to conduct logistic regression and examine the factors associated with treatment acceptance.

To analyze patient survival, the 1-to5-year survival rates of cancer patients were calculated. The Cox proportional hazards model was used to examine the treatment effect and the factors affecting the survival of non-treated patients, after controlling for personal demographics, health status, lung cancer severity, and diagnosing hospital.

## Results

### Demographics

The research population consisted of new lung cancer cases from 2004 to 2008 as provided on the Taiwan Cancer Registry. Overall, 38584 cancer patients had been diagnosed. Among these, 8777 patients refused treatment within 4 months after diagnosis of cancer, accounting for 22.75% of all lung cancer patients.

Bivariate analysis was conducted to compare the treated and non-treated lung cancer patients (Table 1). Variables, including patient age at cancer diagnosis, sex, urbanization level of residence area, insured monthly salary, insurance category, pre-cancer CCI, presence of other catastrophic illnesses, cancer stage, and the accreditation level of diagnosing hospital were significantly different between the 2 groups (*P*<0.001). The proportion of treatment refusals increased with patient age at diagnosis. The average cancer diagnosis age was 73.59±11.50 years for the non-treated patients and 66.16±12.40 years for the treated patients, which was a difference of 7.43±0.90 years.

**Table 1:** Basic demographics and compare the treatment and non-treated lung cancer patients.

Variables	Treatment		Non-treated		P value
	N	%	N	%	
**Total patients**	29807	77.25	8777	22.75	
**Patient age at cancer diagnosis**					<0.001
≤44	1611	89.05	198	10.95	
45–54	4150	89.36	494	10.64	
55–64	6182	87.25	903	12.75	
65–74	9111	79.87	2296	20.13	
≥75	8679	64.12	4856	35.88	
**Average age of cancer diagnosis (M±SD)**	66.16	12.40	73.59	11.50	<0.001
**Gender**					<0.001
Male	19253	76.26	5993	23.74	
Female	10554	79.13	2784	20.87	
**Urbanization level of residence area**					<0.001
Level 1	8130	78.85	2181	21.15	
Level 2	8517	78.84	2286	21.16	
Level 3	4495	76.54	1378	23.46	
Level 4	4672	75.93	1481	24.07	
Level 5	1059	73.44	383	26.56	
Level 6	1500	73.03	554	26.97	
Level 7	1360	73.75	484	26.25	
**Insured monthly salary (US dollars)**					<0.001
Dependent population	8910	76.56	2728	23.44	
≤576	6683	72.35	2554	27.65	
577–760	10032	77.13	2975	22.87	
≥761	4105	89.39	487	10.61	
**Insurance category**					<0.001
Employees/employers	8176	88.47	1066	11.53	
Farmers or fishers	7925	74.56	2704	25.44	
Low-income household	334	71.06	136	28.94	
Unemployed, retired, others	13372	73.30	4871	26.70	
**Pre-cancer CCI**					<0.001
≤3	15615	80.57	3765	19.43	
4–6	5619	76.84	1694	23.16	
7–9	5899	74.64	2004	25.36	
≥10	2674	67.05	1314	32.95	
**Other catastrophic illnesses**					<0.001
No	28838	77.75	8252	22.25	
Yes	969	64.86	525	35.14	
**Lung cancer stage**					<0.001
I	3636	90.90	364	9.10	
II	911	86.60	141	13.40	
III	7770	80.94	1830	19.06	
IV	16210	75.52	5254	24.48	
**Accreditation level of hospital^#^**					<0.001
Medical centers	21168	80.66	5075	19.34	
Regional hospitals	7861	73.25	2871	26.75	
Local hospitals	506	46.81	575	53.19	
Others	119	57.21	89	42.79	
**Hospital ownership^#^**					0.1051
Public hospitals	11025	77.95	3118	22.05	
Private hospitals	18629	77.23	5492	22.77	

Note: CCI, Charlson Comorbidity Index. ^#^The hospital was the diagnosing hospital for non-treatment patients and was the major treatment hospital for treated group.

### Analyses of Factors Related to Patient Treatment Refusal

To avoid cluster effects caused by patients visiting the same hospital, the GEE was conducted with logistic regression model and examined the factors associated with the receiving treatment among lung cancer patients. After controlling for both hospital accreditation level and hospital ownership, several variables, including age ≥65, insured monthly salary between US$ 577 and 760, low-income household and unemployed status (including retirees and others), presence of other catastrophic illnesses, pre-cancer CCI, and cancer stage were all significantly associated with cancer patient treatment refusal (*P*<0.05, Table 2). High treatment refusal rates were associated with older patients, the presence of other catastrophic illnesses, increased pre-cancer CCI score, and advanced cancer stages. Regarding age, patients of ≤44 years of age were used as the reference group. The treatment refusal rate increased for each 10-year age increment. Compared with the reference group, the treatment refusal rate among patients ≥75 years old was increased by 2.61 folds [95% confidence interval (CI) 2.27–2.99].

Concerning socioeconomic status, compared with the reference group (insured salary ≤ US$ 576), the treatment refusal rate among patients with insured salary between US$ 577 and 760 was increased by 1.14 folds (95% CI 1.03–1.26). Compared with the reference group (i.e., employees and employers), based on insurance category, the treatment refusal rate among patients from low-income families was increased by 1.50 folds (95% CI 1.28–1.75). The treatment refusal rate among the unemployed (including retirees and others) patients was increased by 1.26 folds (95% CI 1.15–1.37).

**Table 2:** Analyses of Factors Related to Patient Treatment Refusal (GEE).

Variables	OR	95%CI		P value
**Gender (Male vs. Female)**	0.98	0.92	1.05	0.616
**Age**				
≤44 (reference)				
45–54	0.97	0.87	1.09	0.604
55–64	1.08	0.98	1.19	0.114
65–74	1.45	1.30	1.61	<0.001
≥75	2.61	2.27	2.99	<0.001
**Urbanization level of residence area**				
Level 1 (reference)				
Level 2	0.95	0.89	1.01	0.115
Level 3	1.01	0.95	1.07	0.785
Level 4	0.99	0.92	1.07	0.869
Level 5	1.03	0.92	1.15	0.579
Level 6	1.08	0.93	1.25	0.327
Level 7	1.09	0.98	1.23	0.119
**Insured monthly salary (US dollars)**				
≤576 (reference)				
Dependent population	1.01	0.93	1.09	0.822
577–760	1.14	1.03	1.26	0.013
≥761	1.05	0.94	1.17	0.420
**Insurance category**				
Employees/employers (reference)				
Farmers or fishers	1.03	0.94	1.12	0.568
Low-income household	1.50	1.28	1.75	<0.001
Unemployed, retired, others	1.26	1.15	1.37	<0.001
**Other catastrophic illnesses: Yes vs. No**	1.56	1.40	1.74	<0.001
**Pre-cancer CCI**				
≤3 (reference)				
4–6	1.07	1.02	1.13	0.006
7–9	1.18	1.11	1.25	<0.001
≥10	1.43	1.34	1.53	<0.001
**Lung cancer stage**				
I (reference)				
II	1.21	1.10	1.34	0.001
III	1.57	1.42	1.73	<0.001
IV	2.11	1.87	2.39	<0.001

Note: The model has controlled for diagnosing hospital characteristics including accreditation level of hospital and hospital ownership.

### Relative Risk and Factors Affecting Survival of Lung Cancer Patients

**Figure 1:**
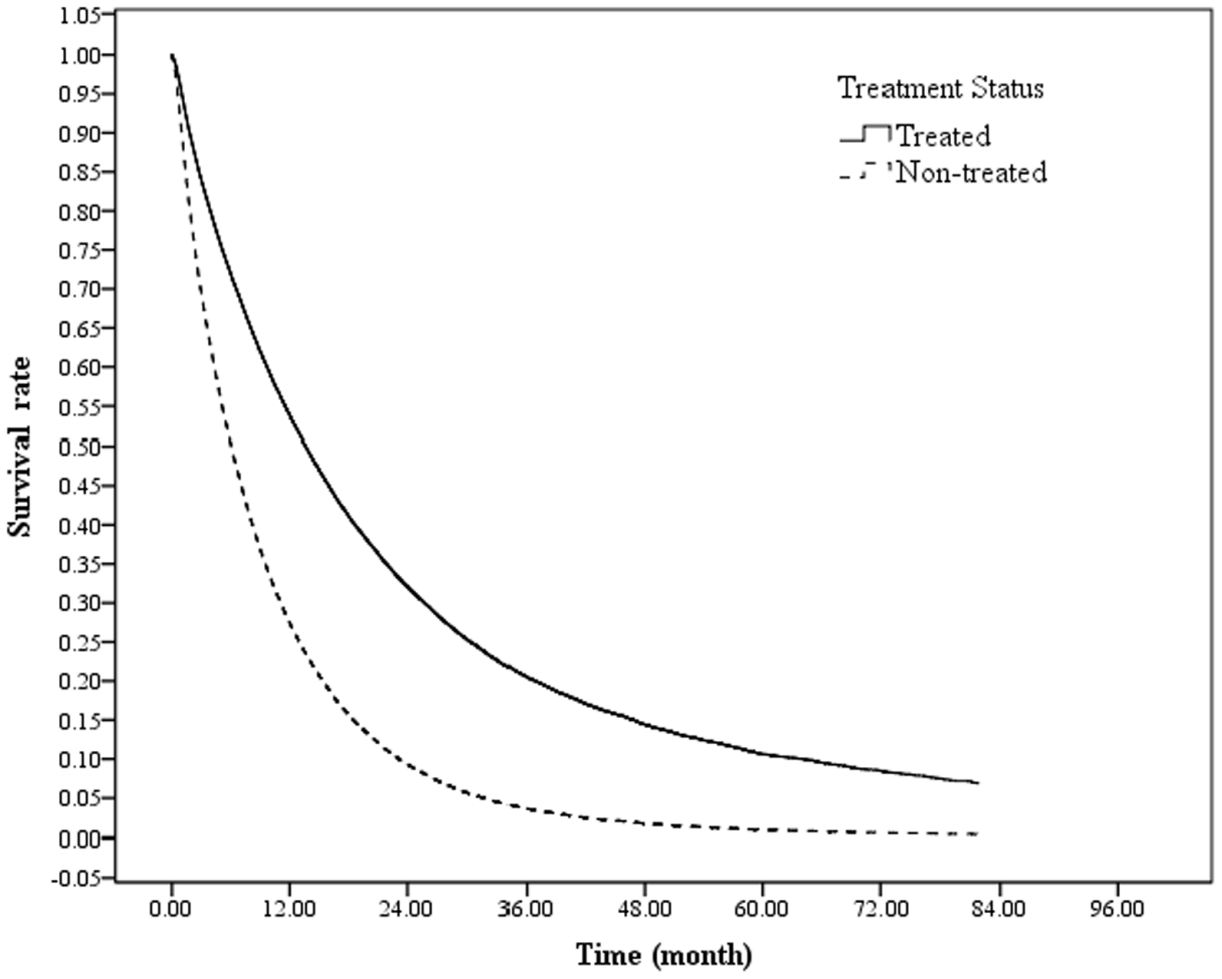
Survival curve using Cox proportional hazards model. After controlling for the other variables, the mortality risk of lung cancer patients refusing treatment was 2.09 times (95% CI 2.03–2.14) that of those receiving treatment.

After controlling for the various variables, compared with lung cancer patients who received treatment, the mortality risk of those refusing treatment was increased by 2.09 folds (95% CI 2.03–2.14, Table 3). Regardless of treatment status, the mortality risk for male lung cancer patients was significantly lower than that for female patients [hazard ratio (HR) 0.66]. An increased mortality rate was also associated with older adults, reduced urbanization levels of residence townships, an insured monthly salary between US$ 577 and 760, low income, unemployment (including retirees and others), presence of other catastrophic illnesses, increased CCI before completing observations, advanced cancer stages, and diagnosed or treated in a private hospital. A comparison of the survival curves of treated and non-treated patients (Fig. 1) indicated that patients receiving treatment exhibited a significantly increased survival rate compared with those without treatment. The survival rates of both groups declined rapidly within 24 months of the lung cancer diagnosis and the survival rate reduction of patients not receiving treatment was more significant than that for treated patients. Furthermore, the 1-year survival rate of treated patients (53.32%) was 31.88% greater than that of non-treated patients (21.44%, Table 4).

**Table 3:** Factors Affecting Survival of Lung Cancer Patients.

Variables	HR	95%CI		P value
**Treatment or not**				
Treatment(reference)				
Non- treated	2.09	2.03	2.14	<0.001
**Gender (Male vs. Female)**	0.66	0.65	0.68	<0.001
**Age**				
≤44 (reference)				
45–54	1.01	0.95	1.08	0.710
55–64	1.05	0.99	1.12	0.100
65–74	1.21	1.14	1.28	<0.001
≥75	1.66	1.56	1.77	<0.001
**Urbanization level of residence area**				
Level 1(reference)				
Level 2	1.00	0.97	1.03	0.876
Level 3	1.05	1.01	1.09	0.006
Level 4	1.02	0.98	1.06	0.266
Level 5	1.01	0.94	1.08	0.813
Level 6	1.13	1.07	1.20	<0.001
Level 7	1.10	1.04	1.16	0.001
**Insured monthly salary (US dollars)**				
≤576 (reference)				
Dependent population	0.99	0.96	1.02	0.436
577–760	1.09	1.02	1.17	0.009
≥761	0.85	0.80	0.91	<0.001
**Insurance category**				
Employees/employers (reference)				
Farmers or fishers	1.01	0.96	1.07	0.761
Low-income household	1.24	1.12	1.39	<0.001
Unemployed, retired, others	1.14	1.08	1.20	<0.001
**Other catastrophic illnesses: Yes vs. No**	1.23	1.16	1.30	<0.001
**CCI before completing observations**				
≤3 (reference)				
4–6	1.72	1.64	1.80	<0.001
7–9	2.04	1.96	2.12	<0.001
≥10	2.02	1.94	2.10	<0.001
**Lung cancer stage**				
I (reference)				
II	1.05	0.96	1.14	0.286
III	1.90	1.82	1.97	<0.001
IV	2.77	2.67	2.87	<0.001
**Hospital ownership**				
Public hospitals (reference)				
Private hospitals	1.21	1.18	1.24	<0.001

Note: The model has controlled for accreditation level of diagnosing hospital.

**Table 4:** The 1-to 5-year survival rates of lung cancer patients.

Survival	Total (%)	Treatment (%)	Non-treated (%)
1 year	46.07	53.32	21.44
2 years	27.96	32.86	11.34
3 years	18.91	22.90	7.62
4 years	14.19	18.29	5.51
5 years	11.16	14.65	4.20

### Analyses of Factors Affecting the Survival of Non-Treated Lung Cancer Patients

The analyses of factors affecting the survival rate of the non-treated lung cancer patients showed that the mortality risk of non-treated male patients was 0.75 times the mortality risk of their female counterparts (95% CI 0.71–0.79, Table 5). Compared with the reference group (patients aged ≤44), the mortality risk of non-treated patients increased with age (HR 1.09–1.71). Compared with the reference group, the mortality risk also increased with the decrease in urbanization level of residence area (HR 0.99–1.18). Compared with the reference group (≤US$ 576), non-treated patients with insured salary of ≥ US$ 761 showed a lower mortality rate. Compared with patients without catastrophic illnesses, those with other catastrophic illnesses exhibited a 1.12-fold increase in mortality risk. In addition, mortality risk increased significantly for those at advanced cancer stages (HR 0.82–1.87). Compared with the reference group (stage I), the mortality risk of non-treated stage IV patients was increased by 1.87 fold (95% CI 1.76–1.99).

**Table 5:** Analyses of Factors Affecting the Survival of Non-Treated Lung Cancer Patients.

Variables	HR	95%CI		Pvalue
**Gender (Male vs. Female)**	0.75	0.71	0.79	<0.001
**Age**				
≤44(reference)				
45–54	1.09	0.91	1.31	0.330
55–64	1.21	1.02	1.43	0.032
65–74	1.38	1.17	1.63	0.001
≥75	1.71	1.45	2.02	<0.001
**Urbanization level of residence area**				
Level 1(reference)				
Level 2	0.99	0.94	1.06	0.853
Level 3	1.08	1.01	1.16	0.034
Level 4	1.07	0.99	1.15	0.095
Level 5	1.06	0.94	1.20	0.363
Level 6	1.13	1.02	1.26	0.022
Level 7	1.18	1.06	1.32	0.003
**Insured monthly salary (US dollars)**				
≤576 (reference)				
Dependent population	0.97	0.91	1.03	0.264
577–760	1.06	0.91	1.23	0.477
≥761	0.77	0.66	0.91	0.002
**Insurance category**				
Employees/employers (reference)				
Farmers or fishers	0.95	0.84	1.08	0.428
Low-income household	1.05	0.85	1.31	0.634
Unemployed, retired, others	1.06	0.93	1.20	0.385
**Other catastrophic illnesses**: Yes vs. No	1.12	1.02	1.22	0.020
**CCI before completing observations**				
≤3 (reference)				
4–6	1.40	1.29	1.52	<0.001
7–9	1.39	1.30	1.49	<0.001
≥10	1.30	1.21	1.39	<0.001
**Lung cancer stage**				
I (reference)				
II	0.82	0.68	0.99	0.037
III	1.36	1.26	1.46	<0.001
IV	1.87	1.76	1.99	<0.001

Note: The model has controlled for diagnosing hospital characteristics including accreditation level of hospital and hospital ownership.

## Discussion

### Analysis of Factors Associated with the Demographics of Patients Refusing Treatment

As shown in Tables 1 and 2, the proportion of patients who refused treatment increased with age. Further analysis by using GEE model revealed an increased treatment refusal rate among elderly patients (*P*<0.001), which was consistent with the results of previous studies [14], [16], [18]–[20]. We presume that elderly patients tend to refuse treatment when being diagnosed with catastrophic and life-threatening diseases because they perceive their lives are ending.

Men exhibited an increased treatment refusal rate compared with women, which concurred with the findings by Chadha et al [21] on treatment refusals of patients at early lung cancer stages (stages I and II). Conversely, Kleffens et al [22] studied 30 cancer patients who refused treatment in the Netherlands and found that increased proportion of men received treatment compared with women. Kleffens et al suggested that the elevated treatment rate was attributable to male family roles, which emphasize financially supporting their families. In this study, further analysis using controlled variables (Table 2) failed to identify significant differences in the probability of non-treatment between men and women in Taiwan (*P*>0.05). This indicated that under the NHI system with a universal coverage, patient decisions to refuse treatment are not associated with sex.

Huchcroft and Snodgrass [23] collected data on non-treated cancer patients in Canada and found that the rate of refusing treatment increased among residents in lowly urbanized areas. Our initial results were similar. The treatment refusal rate increased as the urbanization level of residential areas decreased (Table 1). However, after controlling for the variables by using regression model (Table 2), we found that the urbanization of residential areas was unrelated to patient treatment status, which is inconsistent with previous studies. We speculated that the implementation of the NHI in Taiwan and the promotion of mobile medical programs in rural areas have improved health care access, which is a challenge in Europe and the United States.

Financially, patients categorized as low income exhibited a higher treatment refusal rate (71.06% vs. 28.94%). Further analysis indicated that compared with the reference group (ie, employees and employers), lung cancer patients with low income or who were unemployed (including retirees and others) showed increased treatment refusal rates by 1.26–1.50 folds (95% CI 1.15–1.75, Table 2). Concurrently, Lin, Zhang, and Manson [24] conducted a study on the treatment adherence of breast cancer patients and indicated that medical expenses contributed to treatment non-adherence among cancer patients. Under the NHI provisions and available free access to healthcare for low-income families in Taiwan, treatment refusals remain observable among numerous lung cancer patients. Presumably, health care access is impeded by other factors for low-income families, and other factors may affect treatment decisions, example for close family members support or cancer knowledge [25], [26].

Previous studies have indicated that multiple cancer diagnoses, advanced cancer stages, and health deterioration contribute to treatment refusals or discontinuations among cancer patients [19], [23]. Similarly, our results exhibited an increased treatment refusal rate among patients at advanced cancer stages (HR 1.21–2.11, Table 2). Treatment refusal rates were also increased among patients with elevated pre-cancer CCI scores of comorbidity, those diagnosed with other catastrophic illnesses, and those in poor health. Furthermore, Huchcroft and Snodgrass [19] suggested that although treatment refusals were prevalent among advanced stage cancer patients, numerous other cancer patients refused treatment and further examinations for staging the cancer. However, this portion of treatment refusals is indefinable. Our study experienced a similar challenge, where missing values were observed.

### Examination of Factors Related to Survival among Lung Cancer Patients Refusing Treatment

In this study, compared with patients who received treatment within 4 months after their cancer diagnosis, the mortality risk of those refusing treatment was elevated by 2.09 folds (Table 3). Regardless of treatment status, the mortality risk was lower for male patients than for female patients (HR 0.66, 95% CI 0.65–0.68). Moreover, further analysis on non-treated patients (Table 5) showed that the mortality risk of men who refused treatment within 4 months of cancer diagnosis was 0.75 times lower than that of the mortality risk of women who refused treatment (95% CI 0.71–0.79). These findings were inconsistent with the results of previous studies [27]–[29], which indicated that among lung cancer patients receiving treatment, women showed higher survival and lower mortality risks than did men. Potentially, these inconsistencies are attributable to different drug treatment effects between ethnicities and the sexes.

Furthermore, analysis on factors affecting the survival of cancer patients (Table 3) indicated that increased mortality rates were associated with older patients, diminished urbanization levels of residence area, low income or unemployment (including retirees and others), presence of other catastrophic illnesses, increased CCI, advanced cancer stages, and when the diagnosing hospital was privately owned.

Regarding socioeconomic status, our results supported previous findings [30], [31], showing that young patients with high economic statuses exhibited high survival rates; thus, income inequalities affect patient survival rates.

Moreover, we found that patient mortality risk increased along with the CCI score. Regardless of treatment status, mortality risk was increased in patients at advanced cancer stages. These results supported the findings of previous studies [32]–[34] and confirmed that the CCI and cancer stage are the primary factors affecting cancer patient survival.

Kowalski and Carvalho [35] conducted a study on untreated head and neck cancer and found that nearly 50% of the patients survived for less than 4 months. Chadha et al [21] indicated an average survival period of 11.9 months for untreated patients. In this study, the 1-year survival rate for non-treated lung cancer patients within 4 months of diagnosis was 21.44% (Table 4), whereas the survival rate was 53.32% for treated patients.

As shown in Fig. 1, compared with treated patients, the survival rate of non-treated patients within 4 months of diagnosis was significantly reduced. The average survival time was also reduced for cancer patients at any cancer stage who refused treatment. The 1-year survival rate for treated patients reached 53.32% (Table 4). Compare with the findings of Chadha et al [21], which indicated an average survival time of 11.9 months among non-treated early stage cancer patients. Among the non-treated cancer patients in Taiwan exhibited a higher survival rate. Potentially, the implementation of the NHI, accessible healthcare, and low co-payments for health care have caused this difference. Although Taiwan offers National Health Insurance (NHI), occasionally, cancer patients refuse cancer-related treatment. Potentially, these are attributable to traditional Chinese medicine treatment [36] or Complementary and Alternative medicine [37] in Taiwan.

### Study Limitations

This study had 2 limitations. (1) The 1-to-5 year survival rates of non-treated patients were all significantly lower than those of treated patients. Previous studies [38]–[40] have indicated that in addition to patient demographics, other factors affecting treatment decisions by cancer patients included perceived value of life, the comprehensiveness of the obtained disease information, and religious beliefs. However, these factors were not examined in this study. (2) In this study, patients who received treatment within 4 months of diagnosis were defined as treated patients. Patients who commence treatment after the fifth month were categorized as non-treated. Consequently, the number of non-treated patients could have been overestimated.

## Conclusion

In short, the results indicated that increased treatment refusal rates were associated with men, older adults, reduced urbanization levels of residential areas, reduced insured monthly salary (≤US$ 576), high pre-cancer CCI score, other catastrophic illnesses, and advanced cancer stages. The mortality risk of patients refusing treatment was increased by 2.09 folds (95% CI 2.03–2.14) compared to those receiving treatment. The survival rate of patients not receiving treatment within four months after diagnosis was significant lower than that of treated patients. Analysis after controlling for hospital accreditation levels and ownership indicated that contributing factors to refusing treatment among cancer patients included age, insurance category, the presence of other catastrophic illnesses, pre-cancer CCI score, and cancer stages.

The implementation of the NHI and compulsory enrollment has improved healthcare accessibility and reduced the barriers of obtaining health care. The provisions by the government of mobile medical programs in rural areas and free cancer screenings for specific populations have promoted the early cancer detection and treatment. Despite the low barriers and highly accessible health care environment of lung cancer patients, many patients continue to refuse treatment. Non-treated patients have a significantly reduced survival rate compared with that of treated patients.

These findings can serve as valuable references for policy makers when designing cancer patient care policies and can assist in increasing cancer patient treatment and survival rates.

## Supporting Information

Abbreviations S1 **List of abbreviations.**
(DOCX)
