# Supplementary material for: Factors Associated with Lung Cancer Patients Refusing Treatment and Their Survival: A National Cohort Study under a Universal Health Insurance in Taiwan
Source: PLoS One. 2014 Jul 7;9(7):e101731. doi: 10.1371/journal.pone.0101731 (PMC4084901; doi:10.1371/journal.pone.0101731)
Supplement: Abbreviations S1 — List of abbreviations. (DOCX) [file pone.0101731.s001.docx]

**Supporting information**

NHI : National Health Insurance; CCI: Charlson Comorbidity Index; WHO: World Health Organization; NHIRD: NHI research database; GEE: generalized estimating equation; ANOVA: analysis of variance; CI: confidence interval; HR: hazard ratio.
